# Quantification of bacterial species of the vaginal microbiome in different groups of women, using nucleic acid amplification tests

**DOI:** 10.1186/1471-2180-12-83

**Published:** 2012-05-30

**Authors:** Vicky Jespers, Joris Menten, Hilde Smet, Sabrina Poradosú, Saïd Abdellati, Rita Verhelst, Liselotte Hardy, Anne Buvé, Tania Crucitti

**Affiliations:** 1 Department of Public Health, ITM HIV/AIDS Centre, Institute of Tropical Medicine, Nationalestraat 155, 2000, Antwerp, Belgium; 2 Clinical Trials Unit, Institute of Tropical Medicine, Nationalestraat 155, 2000, Antwerp, Belgium; 3 HIV/STI Reference Laboratory, Institute of Tropical Medicine, Nationalestraat 155, 2000, Antwerp, Belgium; 4 Faculty of Medicine and Health Sciences, Ghent University, De Pintelaan 185, 9000, Ghent, Belgium

## Abstract

**Background:**

The vaginal microbiome plays an important role in urogenital health. Quantitative real time Polymerase Chain Reaction (qPCR) assays for the most prevalent vaginal *Lactobacillus* species and bacterial vaginosis species *G. vaginalis* and *A. vaginae* exist, but qPCR information regarding variation over time is still very limited. We set up qPCR assays for a selection of seven species and defined the temporal variation over three menstrual cycles in a healthy Caucasian population with a normal Nugent score. We also explored differences in qPCR data between these healthy women and an ‘at risk’ clinic population of Caucasian, African and Asian women with and without bacterial vaginosis (BV), as defined by the Nugent score.

**Results:**

Temporal stability of the *Lactobacillus* species counts was high with *L. crispatus* counts of 10^8^ copies/mL and *L. vaginalis* counts of 10^6^ copies/mL. We identified 2 types of ‘normal flora’ and one ‘BV type flora’ with latent class analysis on the combined data of all women. The first group was particularly common in women with a normal Nugent score and was characterized by a high frequency of *L. crispatus*, *L. iners*, *L. jensenii*, and *L. vaginalis* and a correspondingly low frequency of *L. gasseri* and *A. vaginae*. The second group was characterized by the predominance of *L. gasseri* and *L. vaginalis* and was found most commonly in healthy Caucasian women. The third group was commonest in women with a high Nugent score but was also seen in a subset of African and Asian women with a low Nugent score and was characterized by the absence of *Lactobacillus* species (except for *L. iners*) but the presence of *G. vaginalis* and *A. vaginae*.

**Conclusions:**

We have shown that the quantification of specific bacteria by qPCR contributes to a better description of the non-BV vaginal microbiome, but we also demonstrated that differences in populations such as risk and ethnicity also have to be taken into account. We believe that our selection of indicator organisms represents a feasible strategy for the assessment of the vaginal microbiome and could be useful for monitoring the microbiome in safety trials of vaginal products.

## Background

The resident *Lactobacillus* species are the dominant constituents of the healthy vaginal microbiome and play an important role in the defense against sexually transmitted infections (STIs) and HIV [1-3]. Lactobacilli comprise part of the larger innate and adaptive mucosal immune system of the female lower genital tract [4]. The protective mechanisms are still undefined but in addition to the production of lactic acid and the creation of a hostile acid environment, *Lactobacillus* species producing H_2_O_2_ have been shown to inhibit the growth of various micro-organisms, including HIV in vitro [5,6]. Bacterial vaginosis (BV), defined as the colonization of the vagina by several types of anaerobes, including *Gardnerella vaginalis*, together with a reduction in *Lactobacillus* species, has been associated with increased susceptibility to STI and HIV acquisition in both epidemiological studies and in vitro assays [3,6,7].

The findings that alterations in the vaginal microbiome can be associated with negative health outcomes underscores the need for monitoring the composition of the microbiome during trials of vaginal products. The Nugent score is a quick and cheap microscopic tool to assess the presence of *Lactobacillus* species, *G. vaginalisBacteroides* spp. and curved Gram-negative bacilli [8]. Currently this method is considered to be the gold standard for the diagnosis of BV and has been very useful in research but it does not provide reliable identification and quantification of the bacteria at the species level. Molecular techniques based on the amplification of the 16 S ribosomal RNA and 16 S-23 S ribosomal RNA genes from resident bacteria have made it possible to detect and quantify both cultivable and cultivation resistant organisms at the species level [9-11]. Using quantitative real time Polymerase Chain Reaction (qPCR) assays with primers targeting species specific 16 S ribosomal DNA regions, it has been confirmed that a healthy microbiome is dominated by several *Lactobacillus* species [12-15]. Recent pyrosequencing studies suggest that there are a variety of ‘healthy’ microbiomes in the human vagina [14,16]. Ravel et al. proposed five microbiome groups (I to V) in asymptomatic women in the US, distinguishable both by the dominance of *Lactobacillus* species and by the presence of a particular *Lactobacillus* species [14]. Communities in group I are dominated by *L. crispatus*, whereas communities in group II, III, and V are dominated by *L. gasseriL. iners*, and *L. jensenii*, respectively. Communities in group IV are the most diverse and have a higher proportion of strictly anaerobic bacteria in combination with *Lactobacillus* species. Although all five bacterial communities were found in these asymptomatic women, higher Nugent scores were mostly associated with those in group IV.

We set up qPCR assays for the monitoring of the vaginal microbiome during clinical trials of vaginal products based on the following indicator organisms: *Lactobacillus* genus, *L. crispatus, L. iners, L. jensenii, L. gasseriL. vaginalis, Gardnerella vaginalis* and *Atopobium vaginae.* Our aim was to define baseline qPCR values for these bacterial species in a typical healthy population of women not using hormonal contraception and without BV, as defined by the Nugent score, and to describe any temporal variations over 3 menstrual cycles [8,17]. Published data on how quickly the composition of vaginal flora changes are scarce and therefore interpretation of ‘normal’ versus ‘pathological’ in the context of a phase I clinical trial is difficult [18-20]. We also wanted to compare the baseline values in the “healthy population” with available data obtained from a population of women deemed to be “at risk” of STI and HIV on the basis of their attendance at a local low threshold STI and voluntary HIV testing and counseling clinic

## Methods

### Clinical set up

We followed our usual strategy for the recruitment of a classical ‘healthy population’ for phase I microbicide trials [21]. Thirty women were enrolled and followed approximately nine weeks. They were aged between 18 and 35 years, were not using hormonal contraception, did not have vaginal infections at screening, and had a regular menstrual cycle. Any kind of sexual activity was permitted and condoms were provided. After screening, the women received appointments for five follow up visits that were planned on day 7 and 21 of the two next cycles and on day 7 of the third cycle. At each visit the women completed a written questionnaire about their sexual activity during the previous 72 hours.

The second group of women had been recruited six months earlier at a local STI clinic and HIV testing and counseling centre. Women attending the clinic were asked to participate in a study analysing the vaginal microbiome before and after BV treatment. A total of 41 women were enrolled and vaginal samples were taken and tested for STIs and BV on two occasions: at baseline and approximately two weeks later. BV was defined on the basis of a Nugent score of 7 or more and women with BV were treated with a single dose of 2 gram oral metronidazole.

A clinician collected two high vaginal specimens from each woman during every visit, with flocked synthetic swabs (COPAN innovation, Italy). A third vaginal specimen was collected from the healthy women for Prostate Specific Antigen (PSA) testing. The swabs were stored at 2-8 °C and then transported within 12 hours to the laboratory, where they were stored dry at minus 20 °C until testing.

### Laboratory methods

#### DNA extraction

After thawing the swabs at room temperature for 30 minutes, 1200 μL diluted PBS [pH 7.4] (1:9, PBS:saline) was added to the swabs and gently vortexed for at least 15 seconds. The eluates of both swabs were pooled and a final volume of 2000 μL of specimen eluate was obtained. After finalising the samples from the women attending the STI clinic, we learned that DNA yield of Gram positive microorganisms could be improved by adding a lysis step prior to the extraction. This strategy was then applied to the samples of the healthy women and as a result DNA extraction methods differed between the two groups of women. An aliquot of 250 μL eluate of the specimens collected from the healthy population was processed using the easyMag (BioMérieux, Marcy l’Etoile, France) after an initial lysing step with mutanolysin (Sigma Aldrich, Bornem, Belgium) and proteinase K (PK)(Qiagen, Venlo, the Netherlands). Briefly, the aliquot was centrifuged for 10 min at 12500 rpm, and 250 μL mutanolysin/PK buffer was added to the pellet. After vortexing 2.5 μL mutanolysin (25U/μL) was added and incubated for 15 min at 37 °C. Thereafter, a volume of 12.5 μL PK (25 mg/mL) was added and incubated for 15 min at 55 °C including vortexing every 5 minutes. Finally, 1750 μL of Nuclisens Easymag buffer was added prior to the extraction, following the manufacturer’s instructions. For the specimens collected from the clinic population, an aliquot of 500 μL was processed according to the Boom extraction using the miniMAG system (BioMérieux, Marcy l’Etoile, France) and according to the manufacturer’s instructions.

#### Quantitative PCR

Quantitative PCR for total *Lactobacillus* species, *L. crispatus, L. iners, L. jensenii*, *L. gasseri, L. vaginalis, G. vaginalis,* and *A. vaginae* were performed with the primers as described in Table 1. The primers were synthesized by Eurogentec, Seraing, Belgium. The 25 μL PCR mixture contained QuantiTect SYBR Green PCR (Qiagen, Venlo, the Netherlands) with the exception of the PCR mixture for *L. vaginalis* which contained Thermo Scientific Absolute SYBR Green Mix (ABgene, Epsom, UK), 5 μL DNA extract, primers, and Milli-Q water. The amplification reactions were performed using the Corbett Life Science Rotor-Gene™ 6000 (Qiagen, Venlo, the Netherlands) and the amplification programs as described in Table 1. Each sample was run in duplicate. For each of the organisms standard curves were constructed and included in each run. A total of 6 standards were prepared by a tenfold dilution and within a range of 10^2^ copies/5 μL to 10^7^ copies/5 μL. Reference strains (*L. crispatus (*LMG 9479^T^), *L. jensenii* (LMG 6414^T^), *L.iners* (LMG 18914 ^T^), *L. gasseri* (LMG 9203^T^), *L. vaginalis* (LMG^T^ 12891), *G. vaginalis* (LMG 7832 ^T^), *A. vaginae* (CCUG 38953)) were cultured on Columbia agar (Beckton Dickinson, Le pont de Claix, France) supplemented with 5% Defibrinated Horse Blood (E&O laboratories Ltd, Burnhouse, Bonnybridge, Scotland) and incubated in an anaerobic atmosphere (Anaerocult A, Merck Chemicals, Darmstadt, Germany) for 24 hours at 35°C. A suspension was made in 400 μL molecular biology water and DNA was extracted as described above. The DNA concentration was determined by using the Nanodrop ND-1000 (Nanodrop Technnologies, Wilmington, USA). The number of cells in each dilution was calculated taking into account the genome size of the bacterial species. The quantitative result obtained with the qPCR was expressed in number of copies/5 μL and was back calculated taking into account the total specimen elute volume, the volume extracted, the DNA extract volume obtained, and volume of DNA amplified.

**Table 1:** Primers for Quantitative PCR

**PCR**	**Reference**	**Primers**	**Target gene**	**Cycling conditions**	**Concentration**
*L. species*	Zariffard MR [28]	F-LBF: 5′- ATGGAAGAACACCAGTGGCG-3′	16 S r RNA	15 min 95 °C, (15 sec 95 °C, 45 sec 50 °C, 45 sec 72 °C) x37	150 nM
		R- LBR: 5′- CAGCACTGAGAGGCGGAAAC-3′			
*L. crispatus*	Byun R [29]	LcrisF: 5′-AGCGAGCGGAACTAACAGATTTAC-3′	16 S r RNA	15 min, 95 °C, (15 sec 95 °C, 60 sec 60 °C, 20 sec 72 °C) x40	100 nM
		LcrisR : 5′-AGCTGATCATGCGATCTGCTT-3′			
*L. gasseri*	Tamrakar R [30]	LgassF: 5′-AGCGAGCTTGCCTAGATGAATTTG-3′	16 S r RNA	15 min 95 °C, (15 sec 95 °C, 60 sec 57 °C, 60 sec 65 °C) x40	200 nM
		LgassR: 5′-TCTTTTAAACTCTAGACATGCGTC-3′			
*L. iners*	De Backer E [31]	InersFw: 5′-GTCTGCCTTGAAGATCGG-3′	16 S r RNA	15 min 95 °C, (15 sec 95 °C, 55 sec 60 °C, 60 sec 65 °C) x35	200 nM
		InersRev: 5′-ACAGTTGATAGGCATCATC-3′			
*L. jensenii*	Tamrakar R [30]	LjensF: 5′-AAGTCGAGCGAGCTTGCCTATAGA-3′	16 S r RNA	15 min 95 °C, (15 sec 95 °C, 55 sec 60 °C, 60 sec 72 °C) x40	300 nM
		LjensR: 5′-CTTCTTTCATGCGAAAGTAGC-3′			
*L. vaginalis*	In-house designed primers	LV16s_23s_F: 5′-GCCTAACCATTTGGAGGG-3′	16 S-23 S r RNA	15 min 95 °C, (15 sec 95 °C, 30 sec 56 °C, 30 sec 72 °C)x37	200 nM
		LV16s_23s_R3: 5′-CGATGTGTAGGTTTCCG-3′			
*G. vaginalis*	Zariffard MR [28]	F-GV1: 5′-TTACTGGTGTATCACTGTAAGG-3′	16 S r RNA	15 min 95 °C, (45 sec 95 °C, 45 sec 55 °C, 45 sec 72 °C) x50	260 nM
		R-GV3: 5′-CCGTCACAGGCTGAACAGT-3′			
*A. vaginae*	De Backer E [31]	ATOVAGRT3Fw: 5′-GGTGAAGCAGTGGAAACACT-3′ ATOVAGRT3Rev: 5′-ATTCGCTTCTGCTCGCGCA-3′	16 S r RNA	15 min 95 °C, (20 sec 95 °C, 45 sec 60 °C, 45 sec 72 °C) x45	300 nM

#### Prostate specific antigen

The PSA testing was performed using the Seratec® PSA semiquant assay (Seratec Diagnostica, Göttingen, Germany). A volume of 500 μL of PSA buffer was added to the thawed swab and was shaken for 2 hours. After centrifugation of 300 μL for 1 min at 13000 g, 200 μL of supernatant was used for testing, following the manufacturer’s instructions.

#### Data analysis

Baseline characteristics were described using means (ranges) and proportions. We analyzed changes in the profile of the *Lactobacillus* species in the healthy population by defining groups of women based on the consistent presence (present in samples in at least 4 out of 5 visits) or absence of each *Lactobacillus* species. We looked for any predictors of “consistently having a particular species” using logistic regression and predictors of the *Lactobacillus* counts in these women using linear mixed effects models. We compared the presence of individual microbiome species at the baseline visit between ‘healthy population (HP)’ women and ‘clinic population (CP)’ using logistic regression models. We then compared the counts between CP women with (CPBVpos) and without (CPBVneg) bacterial vaginosis using Wilcoxon Rank Sum test. No comparisons in counts between HP and CP species were performed due to the differences in nucleic acid extraction techniques. Using the presence or absence of each of the microbiome species, we divided the study population (CP and HP combined) in groups with Latent Class Analysis, a statistical technique related to cluster analysis, and assessed the distribution of the different groups in the women by BV status and ethnic origin [22]. We assessed the relationship between Nugent scores and the presence of each of the microbiome species in the CP population using scatter plots, and we added a trend-line and a Spearman correlation coefficient R.

#### Ethical approval

IRB approval was obtained from the Institute of Tropical Medicine and from the Ethics Committee at the University Hospital of Antwerp. All study participants gave their written informed consent.

## Results

### Study populations

Baseline characteristics of the two study populations are presented in Table 2. All women recruited into the HP group were Caucasian. They were all asymptomatic at baseline and no diagnosis of BV was made in this group, neither at baseline nor during any of the follow up visits. Five of the 30 HP women (12.5%) had a sexual preference for the same gender and four of them were currently sexually active. Of the remaining 25 heterosexual women, 17 (68%) were currently sexually active. Follow up of the HP women was high, with 28 out of 30 women completing all visits. Prostate specific antigen (PSA) was detected on 12 occasions in 7 women. Of the women recruited at the clinic (CP), 49% were Caucasian, 32% were of black African origin and living in Belgium, 12% of Asian origin, and for 7%, ethnicity was not recorded. 50% percent of the women at the clinic presented with a complaint of vaginal discharge at baseline and 29% had BV as assessed by Nugent score. The presence of self-reported smelly discharge was significantly associated with BV (p = 0.001) but no association was seen between BV and ethnicity.

**Table 2:** Baseline Characteristics of Study Populations

		**Healthy Population (N = 30)**	**Clinic Population**^**a**^**(N = 41)**
			¹
Age (years)	Mean (range)	27 (19–38)	27 (15–47)
			²
Ethnicity N (%)	Black	0 (0)	13 (32)
	Caucasian	30 (100)	20 (49)
	Asian	0 (0)	5 (12)
			³
Contraception N (%)	None	12 (40)	18 (46)
	Combined pill	0 (0)	9 (23)
	Intrauterine device	1 (3)	8 (21)
	Implant	0 (0)	2 (5)
	Condoms	17 (57)	2 (5)
Nugent score 0–3		30 (100%)	29 (71%)
4–6		0 (0%)	0 (0%)
7–10		0 (0%)	12 (29%)

¹ 5 missing values ² 3 missing values ³ 2 missing values.

^a^ STI clinic and HIV testing and counseling centre.

### Changes over time in species presence and species counts in the healthy women

**Figure 1:**
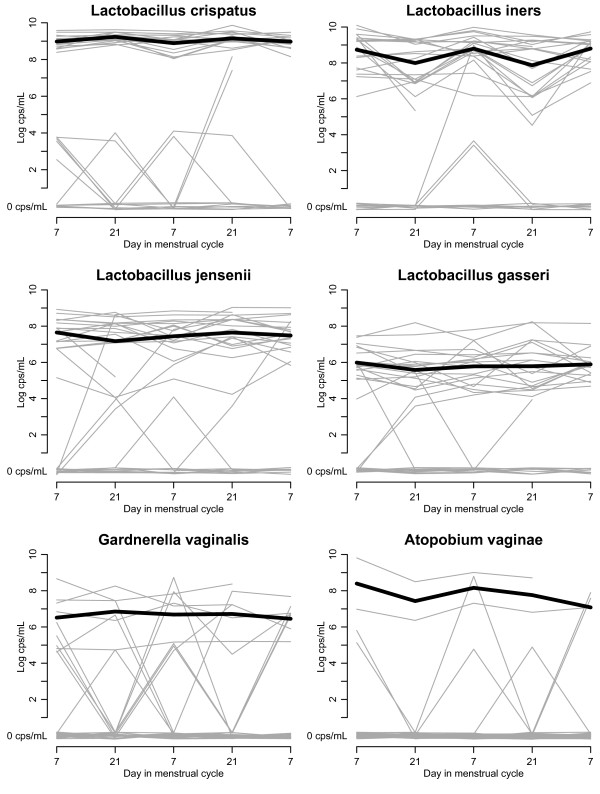
**Presence of species by day in the menstrual cycle.** cps/mL: copies/mL.

In general, the presence or absence of a particular *Lactobacillus* species in the HP remained constant throughout the study visits (Figure 1). *L. crispatus*, *L. iners*, *L. jensenii*, and *L. gasseri* were present at least once in 90%, 77%, 73%, and 70% of women, respectively. *G. vaginalis* was present at least at one visit in 47% of women and *A. vaginae* in 20% of women. *L. crispatus*, *L. iners*, *L. jensenii*, and *L. gasseri* were consistently present (minimum 4 out of 5 visits) in 60%, 67%, 63%, and 67% of women. We categorised the latter group of women, “women with consistent Lactos”. We explored sexual preference; current sexual activity; presence of PSA; time in the menstrual cycle; and age as predictors for being a “women with consistent Lactos”. None of these factors were found to be associated with the consistent presence of lactobacilli. *G. vaginalis* was consistently present in 23% of women and *A. vaginae* in 7% of women. Risk factor analysis was not performed due to low numbers. Longitudinal analysis of the “women with consistent Lactos” showed that *L. crispatus* counts were 0.22 log higher (p < 0.001) and *L. iners* counts were 0.83 log lower (p < 0.001) in the post-ovulatory phase of the cycle. Furthermore, *L. crispatus* counts were decreased by 0.42 log after intercourse (PSA present) (p = 0.002), while those of *L. iners* (+0.73 log, p = 0.033) and of *L. gasseri* (+0.59 log, p = 0.058) were increased.

**Figure 2:**
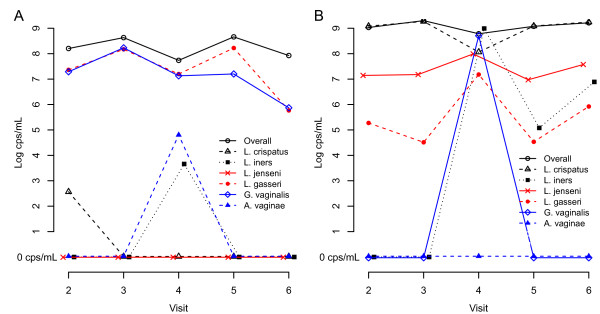
**Presence of species by day in the menstrual cycle for two women developing an elevated Nugent score.** cps/mL: copies/mL.

Two women developed intermediate Nugent scores at visit 4 (6 and 4), while their scores at the other visits were 0. The bacterial cell counts by visit for these two women are shown in Figure 2. In both of these women, the increase in Nugent score coincided with an increase in *L. iners* counts. In the first woman, in whom *G. vaginalis* was present throughout the study, *A. vaginae* appeared on the same day as the raised Nugent score. This woman complained of a vaginal itch and dysuria, had a white watery discharge on examination, and a raised pH of 6.1. In the second woman, *G. vaginalis* appeared together with the elevated Nugent score, while *A. vaginae* remained absent. This woman had a positive PSA test and also had a new sexual partner since the previous visit.

### The vaginal microbiome of the healthy women and the women at risk of STIs

The *Lactobacillus*species were present at baseline in all women. The frequencies of the presence of individual microbiome species are summarized in Table 3, which also presents a pairwise comparison between the HP, the CP without BV (CPBVneg), and the CP with BV (CPBVpos). *L. crispatus* and *L. vaginalis* were significantly more present in HP women and CPBVneg women compared to the CPBVpos women. *L. gasseri* was more often present in HP women compared to the CPBVneg women (p = 0.004), but the differences within the CP were not significant. *L. iners* was less frequently present in the HP compared to the other 2 groups but this was not statistically significant. *G. vaginalis* was significantly more frequently present in CP women than in HP women. *A. vaginae* was significantly more present in CPBVpos compared to HP women and CPBVneg women.

**Table 3:** Presence of species at baseline

	**Healthy population**	**Clinic population**^**a**^		**Pairwise comparisons**		
	BV = 0	BV = 0	BV = 1	HP vs. CPBVneg	HP vs. CPBVpos	CPBVneg vs. CPBVpos
	N = 30	N = 29	N = 12			
	N (%)	N (%)	N (%)	p-value	p-value	p-value
*L. crispatus*	23 (77)	23 (79)	5 (42)	1.000	0.067	0.029
*L. iners*	20 (67)	25 (86)	10 (83)	0.125	0.453	1.000
*L. jensenii*	17 (57)	15 (52)	3 (25)	0.796	0.091	0.171
*L. gasseri*	19 (63)	7 (24)	1 (8)	0.004	0.002	0.214
*L. vaginalis*	22 (73)	18 (62)	1 (8)	0.421	<0.001	0.002
*G. vaginalis*	10 (33)	20 (69)	12 (100)	0.009	<0.001	0.039
*A. vaginae*	4 (13)	8 (28)	11 (92)	0.209	<0.001	<0.001

All P-values from Fisher’s exact test; HP = Healthy population; CPBVneg = Clinic population women without BV; CPBVpos = Clinic population women with BV; vs. =versus; BV = 0 or Nugent scoring 0–3; BV = 1 or Nugent scoring 7–10.

^a^ STI clinic and HIV testing and counseling centre.

When analyzing the presence and absence of microflora species at baseline using Latent Class Analysis (LCA) and combining the ‘healthy population’ and the ‘clinic population’, 3 groups were identified (Table 4). The first group is characterized by the predominance of *L. crispatus*, *L. iners*, *L. jensenii*, and *L. vaginalis* and a low frequency (<30% of women) of *L. gasseri* and *A. vaginae*. This group is mostly prevalent in the women with a normal Nugent score, regardless of whether they belonged to the HP group or to the CP group. The second group is mainly characterized by the presence of *L. gasseri* and *L. vaginalis* and by a less frequent presence of *L. jensenii*, *L. crispatus*, or *L. iners*. This group is mostly prevalent in the Caucasian women, HP women, as well as CP women without BV. The third group is characterized by the presence of *G. vaginalis* and *A. vaginae* and the absence of *Lactobacillus* species, except for *L. iners*. Most women with BV belong to this group, as well as a substantial proportion of African and Asian women without BV.

**Table 4:** Latent class analysis for the presence of species at baseline

**a. Probability (%) of species presence in each of the latent classes**			
	Group 1	Group 2	Group 3
*L. crispatus*	**90**	63	50
*L. iners*	**88**	43	**89**
*L. jensenii*	**84**	24	21
*L. gasseri*	29	**87**	6
*L. vaginalis*	**79**	**70**	16
*G. vaginalis*	50	36	**95**
*A. vaginae*	19	15	**72**
**b. Prevalence (%) of the three latent classes by risk population/BV class**			
	Group 1	Group 2	Group 3
HP	**47**	**47**	6
CP BV neg - Caucasian	**64**	**29**	7
CP BV neg - other	**35**	11	**54**
CP BV pos	9	10	**81**

HP = Healthy population; CPBVneg = Clinic population women without BV; CPBVpos = Clinic population women with BV.

**Figure 3:**
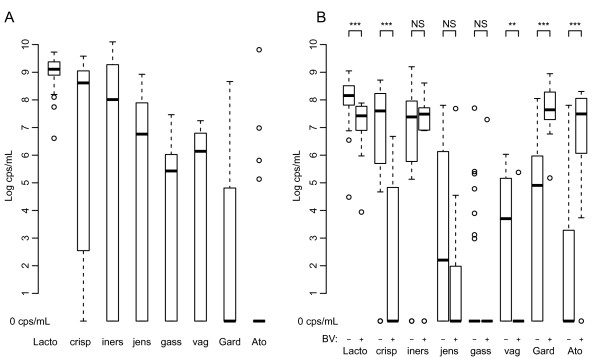
**Presence of species at baseline.***Panel A: Healthy population. Panel B: Clinic population: BV negative* versus *BV positive women.* Lact = Lactobacillus species. crisp = L. crispatus. iners = L. iners. jens = L. jensenii. gass = L. gasseri. vag = L. vaginalis. Gard = G. vaginalis. Ato = A. vaginae. Wilcoxon rank sum test result: ***: p < 0.001; **: p = 0.005; NS: p > 0.100. cps/mL: copies/mL. BV = 0 or Nugent scoring 0–3; BV = 1 or Nugent scoring 7–10.

The qPCR counts are graphically represented in Figure 3. Figure 3 panel B, illustrating the CPBVneg and CPBVpos counts, shows that counts for overall *Lactobacillus* species (p < 0.001), *L. crispatus* (p < 0.001) and *L. vaginalis* (p = 0.005) were significantly higher for women without BV compared to those with BV. The counts for *G. vaginalis* (p < 0.001) and *A. vaginae* (p < 0.001) were, on the contrary, significantly lower in women without BV compared to those with BV. There were no significant differences in the amount of *L. iners, L. gasseri*, and *L. jensenii* related to BV status in the CP.

**Figure 4:**
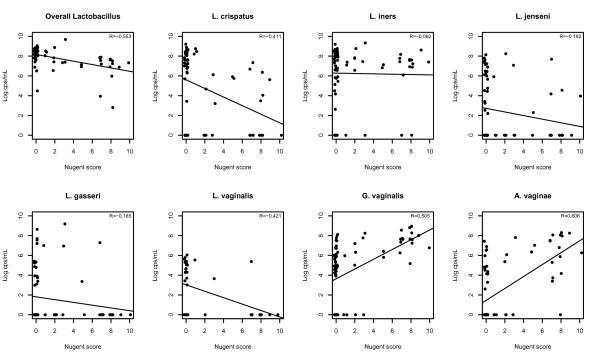
**Correlation of the qPCR log counts data with the individual species by Nugent score.** cps/mL: copies/mL.

The correlation of the qPCR log counts of the individual species of the CP population with the Nugent scores is presented in Figure 4. Overall lactobacillus counts (R = −0.553) and counts of *L. crispatus* (R = −0.411) and *L. vaginalis* (R = −0.421) decreased with increasing Nugent scores. Counts of *G. vaginalis* (R = 0.505) and *A. vaginae* (R = 0.606) increased with increasing Nugent scores. Correlations between Nugent scores and counts of *L. iners* (R = −0.062), *L. jensenii* (R = −0.192), and *L. gasseri* (R = −0.162) were low.

## Discussion

The data from our population of healthy women shows that the composition of the vaginal microbiome over time (5 visits) is very stable. A raised Nugent score (4 and 6) was only recorded on two occasions and we can thus conclude that the microbiome of this population represents a ‘healthy normal flora’.

The increase in *L. crispatus* and the decrease in *L. iners* in the post-ovulatory phase of the menstrual cycle seems in accord with the results of Srinivasan et al., showing a decrease of *L. crispatus* (−0.6 log) during menstruation, followed by a reconstitution of *L. crispatus* after menses [18]. The same authors also noticed that *G. vaginalis* was present for all the women at one point in the study, albeit at low numbers. We found that in 23% of the healthy women, *G. vaginalis* was consistently present. It is interesting to note that in the women from the HP with intermediate Nugent scores, the *L. iners* counts had increased. In the woman with symptoms, this increase was accompanied by a rise in *G. vaginalis* and in the woman with a new sex partner the numbers of *A. vaginae* were raised. Intermediate Nugent scores have been associated with frequent presence of *G. vaginalis* (70% - 92%) and *A. vaginae* (78% - 84%) [23,24]. The acquisition of a new sex partner may well be an important risk factor for BV. Larsson et al. found that relapse of BV in a Swedish population was highly associated (OR 9.3) with the acquisition of a new sex partner and Walker et al. saw that incident BV in Australian young women was associated with increasing numbers of sex partners [23,25].

Using LCA, we identified 2 types of ‘normal flora’ and one ‘BV type flora’. The first group of ‘normal flora’ was characterized by the predominance of a combination of four *Lactobacillus* species excluding *L. gasseri,* whereas in the second group *L. gasseri* and *L. vaginalis* predominated. The third group, associated with BV, was dominated by *A. vaginae, G. vaginalis*, and *L. iners.* Group 1 in our study was similar to community groups I, III, and V as defined by Ravel et al.; group 2 corresponded to community group II, and group 3 was similar to community group IV [14]. All 3 microbiome groups were represented in the different groups of women (HP, CP without BV, and CP with BV). However, among the women without BV there appeared to be large differences in the relative distribution of the different LCA groups according to ethnicity. Caucasian women mostly belonged to group 1 or 2, while African/Asian women mostly belonged to group 3. We should therefore not assume that all microbiomes with low Nugent scores are similar. Our data are in line with the findings of Ravel et al., who reported that healthy African/Asian women have a higher probability of belonging to group 3, the ‘BV type flora’ group [16,26].

The results of this study are in line with published literature showing that *L. crispatus* is consistently present with high counts of >10^8^ copies/mL in a healthy vaginal ecosystem as defined by the Nugent score (0–3) whereas *G. vaginalis* and *A. vaginae* are highly present in women with BV [11,24]. We explored the correlation of specific species with the individual Nugent scores and showed that *L. vaginalis* (R = −0.421) shows the same inverse correlation as *L. crispatus* (R = −0.411) with increasing Nugent scores. A low correlation was seen for *L. gasseri* and the Nugent score and this may reflect the confounding effect of ethnicity. This study is among the first to show that *L. vaginalis* is highly represented in the normal healthy vaginal flora with typical counts of 10^6^ copies/mL. *L. crispatus, L. jensenii, L. gasseri,* and *L. vaginalis* were less frequently present in women at higher risk of an STI, while *L. iners* remained present. The fact that *L. iners* is always present*,* even when *A. vaginae* and *G. vaginalis* are present, makes us wonder whether *L. iners* increases susceptibility to BV. This would be in line with the findings of Antonio et al. who recently demonstrated that only *L. crispatus* had a protective effect against acquisition of BV [27].

We observed higher bacterial counts with the combined lysis-Boom extraction compared to the Boom extraction alone (results not shown). The extra lysis step particularly improved the efficiency of the DNA extraction from Gram positive microorganisms. As a result of these different methods of extraction, we were unable to directly compare the quantitative counts from the HP and CP group (Figure 3) and this represents a weakness of this study. This shortcoming illustrates that results across studies can only be compared after ascertaining that laboratory methods are consistent [28,29]. Another limitation of this study is the small sample size and limited statistical power. Furthermore, the two groups of women differed in aspects such as contraception, the number of follow up visits and time points in the cycle that were sampled. Finally, our definition of bacterial vaginosis was based on the Nugent score, and although this scoring system is considered to be the gold standard for research, we recognize it is not perfect.

## Conclusion

We have shown that qPCR can be used to quantify and describe the bacterial species associated with the non-BV vaginal microbiome. We have also shown that risk status and ethnicity can also impact upon the number and type of organisms present and therefore also need to be taken into account. The analysis of seven indicator organisms by qPCR is a feasible approach for the assessment of the vaginal microbiome and could be used for analyzing the composition of the microbiome during the safety assessments of vaginal products.

## Authors’ contributions

VJ, TC, and AB conceived and designed the study. VJ wrote the first version of the manuscript. JM provided statistical support for the design of the study and performed the statistical analyses. TC supervised the laboratory analytical procedures and validated the laboratory results. TC, HS, SA and RV set up and carried out the qPCRs. SP and LH participated in the design and clinical coordination of the study. All authors contributed to the editing, and approved the final paper.

## References

[B1] Myer L Kuhn L Stein ZA Wright TC Denny L Intravaginal practices, bacterial vaginosis, and women’s susceptibility to HIV infection: epidemiological evidence and biological mechanisms Lancet Infect Dis 2005 5 786 794 10.1016/S1473-3099(05)70298-X 16310150

[B2] Taha TE Hoover DR Dallabetta GA Kumwenda NI Mtimavalye LA Yang LP Liomba GN Broadhead RL Chiphangwi JD Miotti PG Bacterial vaginosis and disturbances of vaginal flora: association with increased acquisition of HIV AIDS 1998 12 1699 1706 10.1097/00002030-199813000-00019 9764791

[B3] van de Wijgert JH Morrison CS Brown J Kwok C Van Der PB Chipato T Byamugisha JK Padian N Salata RA Disentangling contributions of reproductive tract infections to HIV acquisition in African Women Sex Transm Dis 2009 36 357 364 10.1097/OLQ.0b013e3181a4f695 19434010

[B4] Mirmonsef P Gilbert D Zariffard MR Hamaker BR Kaur A Landay AL Spear GT The effects of commensal bacteria on innate immune responses in the female genital tract Am J Reprod Immunol 2011 65 190 195 10.1111/j.1600-0897.2010.00943.x 21143335 PMC3581076

[B5] Hillier SL Krohn MA Rabe LK Klebanoff SJ Eschenbach DA The normal vaginal flora, H2O2-producing lactobacilli, and bacterial vaginosis in pregnant women Clin Infect Dis 1993 16 Suppl 4 S273 S281 8324131 10.1093/clinids/16.supplement_4.s273

[B6] Klebanoff SJ Coombs RW Viricidal effect of Lactobacillus acidophilus on human immunodeficiency virus type 1: possible role in heterosexual transmission J Exp Med 1991 174 289 292 10.1084/jem.174.1.289 1647436 PMC2118880

[B7] Cherpes TL Hillier SL Meyn LA Busch JL Krohn MA A delicate balance: risk factors for acquisition of bacterial vaginosis include sexual activity, absence of hydrogen peroxide-producing lactobacilli, black race, and positive herpes simplex virus type 2 serology Sex Transm Dis 2008 35 78 83 10.1097/OLQ.0b013e318156a5d0 17989585

[B8] Nugent R Krohn M Hillier S Reliability of diagnosing bacterial vaginosis is improved by a standardized method of gram stain interpretation J Clin Microbiol 1991 29 297 301 1706728 10.1128/jcm.29.2.297-301.1991 PMC269757

[B9] Hugenholtz P Goebel BM Pace NR Impact of culture-independent studies on the emerging phylogenetic view of bacterial diversity J Bacteriol 1998 180 4765 4774 9733676 10.1128/jb.180.18.4765-4774.1998 PMC107498

[B10] Sha BE Chen HY Wang QJ Zariffard MR Cohen MH Spear GT Utility of Amsel criteria, Nugent score, and quantitative PCR for Gardnerella vaginalis, Mycoplasma hominis, and Lactobacillus spp. for diagnosis of bacterial vaginosis in human immunodeficiency virus-infected women J Clin Microbiol 2005 43 4607 4612 10.1128/JCM.43.9.4607-4612.2005 16145114 PMC1234056

[B11] Verhelst R Verstraelen H Claeys G Verschraegen G Delanghe J Van Simaey L De Ganck C Temmerman M Vaneechoutte M Cloning of 16 S rRNA genes amplified from normal and disturbed vaginal microflora suggests a strong association between Atopobium vaginae, Gardnerella vaginalis and bacterial vaginosis BMC Microbiol 2004 4 16 10.1186/1471-2180-4-16 15102329 PMC419343

[B12] Fredricks DN Fiedler TL Thomas KK Oakley BB Marrazzo JM Targeted PCR for detection of vaginal bacteria associated with bacterial vaginosis J Clin Microbiol 2007 45 3270 3276 10.1128/JCM.01272-07 17687006 PMC2045326

[B13] Hummelen R Fernandes AD Macklaim JM Dickson RJ Changalucha J Gloor GB Reid G Deep sequencing of the vaginal microbiota of women with HIV PLoS One 2010 5 e12078 10.1371/journal.pone.0012078 20711427 PMC2920804

[B14] Ravel J Gajer P Abdo Z Schneider GM Koenig SS McCulle SL Karlebach S Gorle R Russell J Tacket CO Brotman RM Davis CC Ault K Peralta L Forney LJ Vaginal microbiome of reproductive-age women Proc Natl Acad Sci USA 2011 108 Suppl 1 4680 4687 20534435 10.1073/pnas.1002611107 PMC3063603

[B15] Spear GT Gilbert D Landay AL Zariffard R French AL Patel P Gillevet PM Pyrosequencing of the genital microbiotas of HIV-seropositive and -seronegative women reveals Lactobacillus iners as the predominant Lactobacillus Species Appl Environ Microbiol 2011 77 378 381 10.1128/AEM.00973-10 21075899 PMC3019699

[B16] Zhou X Brown CJ Abdo Z Davis CC Hansmann MA Joyce P Foster JA Forney LJ Differences in the composition of vaginal microbial communities found in healthy Caucasian and black women ISME J 2007 1 121 133 10.1038/ismej.2007.12 18043622

[B17] Lamont R Sobel J Akins R Hassan S Chaiworapongsa T Kusanovic J Romero R The vaginal microbiome: new information about genital tract flora using molecular based techniques BJOG 2011 118 533 549 10.1111/j.1471-0528.2010.02840.x 21251190 PMC3055920

[B18] Srinivasan S Liu C Mitchell CM Fiedler TL Thomas KK Agnew KJ Marrazzo JM Fredricks DN Temporal variability of human vaginal bacteria and relationship with bacterial vaginosis PLoS One 2010 5 e10197 10.1371/journal.pone.0010197 20419168 PMC2855365

[B19] Verstraelen H Verhelst R Claeys G De Backer E Temmerman M Vaneechoutte M Longitudinal analysis of the vaginal microflora in pregnancy suggests that L. crispatus promotes the stability of the normal vaginal microflora and that L. gasseri and/or L. iners are more conducive to the occurrence of abnormal vaginal microflora BMC Microbiol 2009 9 116 10.1186/1471-2180-9-116 19490622 PMC2698831

[B20] Santiago GL Cools P Verstraelen H Trog M Missine G El Aila N Verhelst R Tency I Claeys G Temmerman M Vaneechoutte M Longitudinal study of the dynamics of vaginal microflora during two consecutive menstrual cycles PLoS One 2011 6 e28180 10.1371/journal.pone.0028180 22140538 PMC3227645

[B21] Jespers VA Van Roey JM Beets GI Buve AM Dose-ranging phase 1 study of TMC120, a promising vaginal microbicide, in HIV-negative and HIV-positive female volunteers J Acquir Immune Defic Syndr 2007 44 154 158 10.1097/QAI.0b013e31802bb35f 17106275

[B22] McCutcheon AL Latent Class Analysis. Quantitative Applications in the Social Sciences Series N° 64 1987 Sage Publication, Thousand Oaks edition

[B23] Larsson PG Brandsborg E Forsum U Pendharkar S Krogh-Andersen K Nasic S Hammarstrom L Marcotte H Extended antimicrobial treatment of bacterial vaginosis combined with human lactobacilli to find the best treatment and minimize the risk of relapses BMC Infect Dis 2011 11 223 10.1186/1471-2334-11-223 21854593 PMC3176208

[B24] Menard JP Fenollar F Henry M Bretelle F Raoult D Molecular quantification of Gardnerella vaginalis and Atopobium vaginae loads to predict bacterial vaginosis Clin Infect Dis 2008 47 33 43 10.1086/588661 18513147

[B25] Walker J Hocking J Fairley C Tabrizi S Chen M Bowden F Gunn J Donovan B Kaldor J Bradshaw C The prevalence and incidence of bacterial vaginosis in a cohort of young Australian women Sex Transm Infect 2011 Vol 87 Suppl 1

[B26] Zhou X Hansmann MA Davis CC Suzuki H Brown CJ Schutte U Pierson JD Forney LJ The vaginal bacterial communities of Japanese women resemble those of women in other racial groups FEMS Immunol Med Microbiol 2010 58 169 181 10.1111/j.1574-695X.2009.00618.x 19912342 PMC2868947

[B27] Antonio M Petrina M Meyn L Hillier S Lactobacillus crispatus colonisation reduces risk of bacterial vaginosis (BV) acquisition Sex Transm Dis 2011 Vol 87 Suppl 1 A304 A305

[B28] Zariffard MR Saifuddin M Sha BE Spear GT Detection of bacterial vaginosis-related organisms by real-time PCR for Lactobacilli, Gardnerella vaginalis and Mycoplasma hominis FEMS Immunol Med Microbiol 2002 34 277 281 10.1111/j.1574-695X.2002.tb00634.x 12443827

[B29] Byun R Nadkarni MA Chhour KL Martin FE Jacques NA Hunter N Quantitative analysis of diverse Lactobacillus species present in advanced dental caries J Clin Microbiol 2004 42 3128 3136 10.1128/JCM.42.7.3128-3136.2004 15243071 PMC446321

[B30] Tamrakar R Yamada T Furuta I Cho K Morikawa M Yamada H Sakuragi N Minakami H Association between Lactobacillus species and bacterial vaginosis-related bacteria, and bacterial vaginosis scores in pregnant Japanese women BMC Infect Dis 2007 7 128 10.1186/1471-2334-7-128 17986357 PMC2212641

[B31] De Backer E Verhelst R Verstraelen H Alqumber MA Burton JP Tagg JR Temmerman M Vaneechoutte M Quantitative determination by real-time PCR of four vaginal Lactobacillus species. Gardnerella vaginalis and Atopobium vaginae indicates an inverse relationship between L. gasseri and L. iners BMC Microbiol 2007 7 115 10.1186/1471-2180-7-115 18093311 PMC2233628

